# Integrated metabolomic and transcriptomic analyses revealed metabolite variations and regulatory networks in *Cinnamomum cassia* Presl from four growth years

**DOI:** 10.3389/fpls.2023.1325961

**Published:** 2024-01-10

**Authors:** Hongyang Gao, Min Shi, Huiju Zhang, Hongli Shang, Quan Yang

**Affiliations:** ^1^ School of Chinese Materia Medica, Guangdong Pharmaceutical University, Guangzhou, China; ^2^ Guangdong Provincial Research Center on Good Agricultural Practice & Comprehensive Agricultural Development Engineering Technology of Cantonese Medicinal Materials, Guangzhou, Guangdong, China; ^3^ Comprehensive Experimental Station of Guangzhou, Chinese Material Medica, China Agriculture Research System (CARS-21-16), Guangzhou, China; ^4^ Key Laboratory of State Administration of Traditional Chinese Medicine for Production & Development of Cantonese Medicinal Materials, Guangzhou, Guangdong, China

**Keywords:** *Cinnamomum cassia* Presl, phenylpropanoids, flavonoids, terpenoids, transcriptome

## Abstract

To understand the mechanism of the dynamic accumulation of active ingredients in *Cinnamomum cassia* Presl, metabolomic and transcriptomic analyses of 5~8 years old *C*. *cassia* were performed. A total of 72 phenylpropanoids, 146 flavonoids, and 130 terpenoids showed marked changes. Most phenylpropanoids and flavonoids showed markedly higher abundances in 6-year-old *C*. *cassia* than in others, which was related to the higher expression of genes that synthesize and regulate phenylpropanoids and flavonoid. We identified transcription factors (TFs) and genes involved in phenylpropanoids and flavonoids synthesis and regulation through co-expression network analyses. Furthermore, most of the terpenoids in 5-year-old *C*. *cassia* showed markedly higher abundances than in others, which was due to the differentially expressed genes upstream of the terpenoids pathway. The results of our study provide new insights into the synthesis and accumulation of phenylpropanoid, flavonoids and terpenoids in *C*. *cassia* at four growth stages.

## 1 Introduction

*Cinnamomum cassia* Presl is a perennial arborous plant of the Lauraceae family, which is an important cash crop in many countries in the world and is widely used in many fields, such as chemical industry, food, and medicine (Jeyaratnam et al., 2016). The bark of *C*. *cassia*, an important traditional medicinal and edible plants, is often used as a spice to add flavor and aroma to food. It also has anti-inflammatory, hypoglycemic, anti-oxidant, anti-tumor and other pharmacological activities (Koppikar et al., 2010; Shin et al., 2017; Kang and Lee, 2018). In addition, cinnamon essential oil has a broad antibacterial spectrum, can inhibit foodborne pathogens and putrefactive bacteria (Vijayan and Mazumder, 2018).The edible film made by combining with oxidized hydroxypropyl cassava starch has better performance and can be used as packaging materials for fruits and vegetables and food, which can inhibit the pollution of foodborne pathogens and spoilage bacteria and extend the shelf life of food (Zhang et al., 2016; Zhou et al., 2021).

*C*. *cassia* has a long growth cycle, which requires at least 4–6 years of growth, sometimes even decades of growth. The bark of *C*. *cassia* often harvested from 5~8-year-old trees. In recent years, the demand for *C*. *cassia* in the international market has increased, leading to differences in the harvesting period of *C*. *cassia* and affecting its quality. The main active component of *C*. *cassia* is volatile oil, which consists of cinnamaldehyde, cinnamic acid, coumarin, sesquiterpene, and diterpene. In addition, *C*. *cassia* contains flavonoids, anthocyanins, and other non-volatile components. Among them, cinnamaldehyde, cinnamic acid, coumarin, flavonoids, anthocyanins, and other substances are directly or indirectly produced through phenylpropanoid biosynthesis (Fraser and Chapple, 2011). Terpenoids are synthesized by two different metabolic pathways: the mevalonate (MVA) pathway and the 2-c-methyl-d-erythritol 4-phosphate (MEP) pathway. The composition and content of volatile oil in *C*. *cassia* are affected by growth years and other factors (Geng et al., 2011). Li et al. (2013) studied the development of oil cells in *C*. *cassia* leaves of different ages and found that the density of oil cells in leaves of 2-year-old branches was the highest, which directly affected the content of cinnamaldehyde. In addition, Geng et al. (2011) measured the content and composition of volatile oil of *C*. *cassia* aged from 1 to 12 years and found that the yield and composition fluctuated at each development stage, with the situation first increasing and then decreasing. The cinnamaldehyde content in 6-year-old *C*. *cassia* is the highest, but its molecular mechanism has not been clarified. In the past, most research on *C*. *cassia* with different growth years has focused on the differences in chemical components, while research on the synthesis pathway and molecular regulation of effective components in *C*. *cassia* with different growth years has not been carried out.

At present, integrative analysis of metabolome and transcriptome has been successfully applied to the study of synthesis and regulatory mechanisms of active ingredients in plant. The molecular mechanism of different accumulations of phenylpropanoids, flavonoids, and terpenoids in *Ginkgo biloba* was systematically studied by metabonomics and transcriptomics, and the expression levels of related synthetic genes and regulatory effects of transcription factors (TFs) were analyzed (Meng et al., 2019; Guo et al., 2020). At the same time, researchers have successfully revealed the biological molecular mechanism of effective substance synthesis in *Carthamus tinctorius*, *Dendrobium officinale, Lonicera japonica* Thunb and other plants through integrative analysis of transcriptome and metabolome (Xue et al., 2019; Wang et al., 2021; Li et al., 2022). Although previous studies have used transcriptomics and metabolomics to analyze metabolites and genes in different *C*. *cassia* tissues. The content differences of active components such as active flavonoids in bark, branches and leaves of *C*. *cassia* were revealed, and the differentially expressed genes that may affect the synthesis of active components in cinnamon were identified (Gao et al., 2023). However, there is a lack of extensive and comprehensive research on the synthesis of effective substances in *C*. *cassia* with different growth years.

This study systematically analyzed the differences in gene expression and metabolism between the bark of *C*. *cassia* aged 5~8 years. Integrative analysis of metabolome and transcriptome were used to study the correlation between these DEGs (differentially expressed genes), TFs and DAMs (differentially accumulated metabolites) in the synthesis pathway of phenylpropanoids, flavonoids, and terpenoids. The results provided theoretical basis for studying the internal mechanism of effective component accumulation and quality formation of *C*. *cassia* and lay a foundation for efficient cultivation of *C*. *cassia* and increase the yield of volatile oil of cinnamon.

## 2 Materials and methods

### 2.1 Plant materials

*C*. *cassia*, aged 5, 6, 7, and 8 years, collected from Sili Village, Tanbin Town, Yunfu City, Guangdong Province (22°50’52”N, 111°24’35”E), were used in this experiment, and the selected trees had the same cultivation and management conditions. On October 25, 2019, we peeled the bark of 5~8-year-old *C*. *cassia* about 1 m above the ground, and the collected bark then stored in a −80°C refrigerator for a maximum of a week.

### 2.2 Metabolite extraction and profiling

Organic reagents were used to extract metabolites from the cinnamon samples. From samples of the same year, 50 µL of filtered extract was mixed as a QC sample. Non-targeted metabonomic analysis based on liquid chromatography-tandem mass spectrometry was used to detect metabolites in 5~8-year-old samples. Six replicates were made for each sample. Chromatographic analysis was performed with ACQUITY UPLC HSS T3 column (Waters). The column temperature was set at 50°C and 5 µL was injected each time. Water containing 0.1% formic acid and methanol containing 0.1% formic acid were used as mobile phase for gradient elution at a flow rate of 0.4ml/min. The products eluted from the chromatographic column were collected using a mass spectrometer Xevo G2-XS QTOF (Waters, UK) in both positive and negative ion detection modes.

PCA and PLS-DA were used to determine the metabonomic differences among 5~8-year-old samples, and the DAMs were screened based on the conditions of VIP ≥ 1, q-value<0.05 and fold change ≥ 1.2 or ≤ 0.8333. The DAMs between the comparison groups were annotated into the corresponding pathway in the KEGG database, and the significant enrichment pathways of metabolites were screened. TBtools 1.098 was used to create metabolite intensity heatmaps.

### 2.3 RNA extraction and transcriptome analysis

Using the plant total RNA extraction kit (TIANGEN) to extract RNA. After purification and fragmentation, it was reverse transcribed into cDNA, and then terminal repair was performed. Finally, the poly(A) tail and adaptor was added for PCR amplification and the DNA library was obtained after the amplification product was purified. Deep sequencing of the transcriptome was then carried out on the BGISEQ-500 sequencing platform of the BGI Gene.

SOAPnuke 1.4.0 was used to filter out reads containing low-quality, contaminated joints, and high levels of unknown base N from the raw data obtained from machine sequencing. Then use Bowtie2 2.2.5 (http://bowtie-bio.sourceforge.net/bowtie2/index.shtml) to compare clean reads to the reference gene sequence. Assembled clean reads using Trinity 2.0.6 software to obtain Unigenes. The Unigenes were compared with seven functional databases NR (ftp://ftp.ncbi.nlm.nih.gov/blast/db), NT (ftp://ftp.ncbi.nlm.nih.gov/blast/db), SwissProt (http://www.expasy.ch/sprot/), KEGG (http://www.genome.jp/kegg), KOG (https://www.ncbi.nlm.nih.gov/COG/), Pfam (http://pfam.xfam.org) and GO (http://geneontology.org) for annotations. RSEM 1.2.8 software was used to calculate the Fragments Per Kilobase Million (FPKM) value of expression, and FPKM > 0.3 was considered differential expression. The difference of gene expression of *C*. *cassia* in each year was analyzed with 5-year olds as the control. Use DEseq2 to screen for differentially expressed genes, with the screening condition set to q-value ≤ 0.05. Then KEGG enrichment analysis was performed and q-value ≤ 0.05 was considered as significant enrichment. The detailed transcriptome data has been submitted to the NCBI Public Library, with the Sequence Read Archive (SRA) number PRJNA1041972.

### 2.4 qRT-PCR analysis

Twelve DEGs on the pathway of flavonoids, phenylpropanoids, and terpenoids were selected for qRT-PCR validation. According to the sequence obtained, primers were designed using Primer Quest (**Supplementary Table S1**), and TB Green® Premix Ex Taq™ II (Takara) was used to conduct qRT-PCR. The thermal cycling conditions were as follows: pre-denaturation at 95°C for 30 s, followed by 40 cycles of 95°C for 5 s and 59°C for 30 s. The melting curve was formed to evaluate the specificity of the expansion product, and the gene expression were calculated by 2^-ΔΔCT^.

### 2.5 Integrative analysis of metabolome and transcriptome

Based on the annotation results of DAMs and DEGs on the KEGG pathway, the gene FPKM values and metabolite intensity in each age group of cinnamon samples were Z-score standardized and a heatmap was drawn. The correlation between DAMs, TFs, and DEGs were calculated using the Pearson correlation coefficient method, with screening conditions of Pearson correlation coefficient >| 0.8 |, P value<0.05. Then Cytoscape 3.7.1 was used to map the network relationship.

## 3 Results

### 3.1 Metabonomic analysis of 5~8-year-old C. cassia

**Figure 1:**
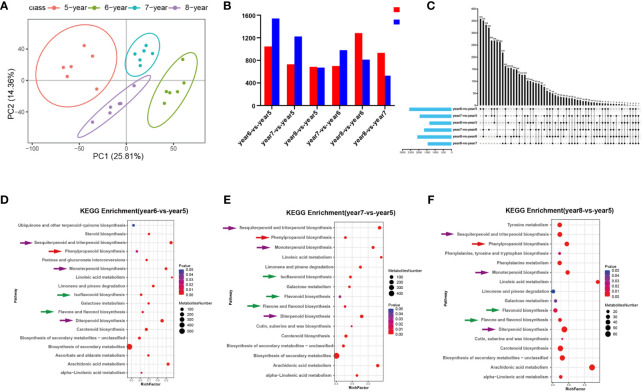
Statistical analysis of metabonomic data of 5~8-year-old *C. cassia*. **(A)** Principal component analysis diagram of test sample and quality control sample in negative ion mode. **(B)** Comparing 5~8-year-old *C. cassia* in pairs to obtain the number of different metabolites upregulated and downregulated in each group. **(C)** Upstet Plot set diagram of the different metabolites. The left histogram shows the total number of DAMs included in each group comparison. The lower part of the intersection point represents the corresponding comparison group on the left, and the bar graph on the top represents the amount of DAMs shared under the intersection condition. **(D)** KEGG enrichment analysis of DAMs in year6-*vs.*-year5. **(E)** KEGG enrichment analysis of DAMs in year7-*vs.*-year5. **(F)** KEGG enrichment analysis of DAMs in year8-*vs.*-year5.

*C*. *cassia* samples aged 5~8 years were analyzed using UPLC-MS/MS, and ions with RSD ≤ 30% were selected for subsequent analysis. PCA and heatmap cluster analysis showed that there were significant differences between 5~8-year-old samples, indicating that growth years had a greater impact on the accumulation of effective metabolites in *C*. *cassia* (**Figure 1A**; **Supplementary Figure S1**). The 5-year-old samples were used as controls to screen the DAMs. Among them, there were 2,586 metabolites with significant differences in year6-*vs.*-year5, of which 1,045 increased and 1,541 decreased. In year 7-*vs.*-year 5, 1952 DAMs were screened, of which 730 increased and 1,222 decreased. In year8-*vs.*-year5, there were 1,357 different metabolites, of which 685 increased and 672 decreased (**Figure 1B**). There were 359, 334, 156, 322, 262, and 163 unique DAMs in the year6-*vs.*-year5, year7-*vs.*-year5, year7-*vs.*-year6, year8-*vs.*-year6, and year8-*vs.*-year7 comparison groups, respectively (**Figure 1C**). The screened DAMs from year6-*vs.*-year5, year7-*vs.*-year5, and year8-*vs.*-year5 were analyzed for KEGG pathway enrichment, and when Qvalue ≤ 0.05 it was considered as significant enrichment. The results indicated that the three groups of DAMs were significantly enriched in biosynthetic pathways of phenylpropanoid, flavonoid, flavone and flavonol, isoflavonoid, monoterpenoid, diterpenoid, sesquiterpenoid and triterpenoid (**Figures 1D–F**). This indicates that the accumulation of phenylpropanoids, flavonoids, and terpenoids in *C*. *cassia* changed with the growth years.

### 3.2 DAMs in 5~8-year-old C. cassia

**Figure 2:**
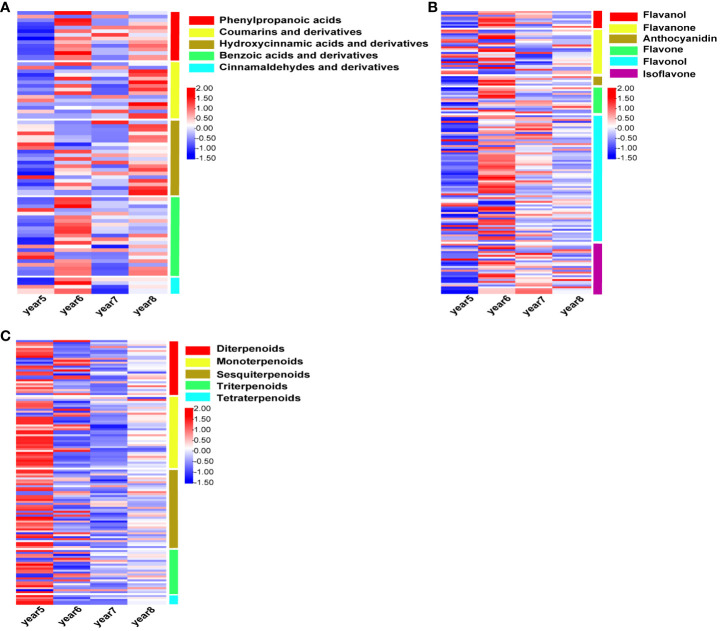
Heat map of intensity of phenylpropanoids **(A)**, flavonoids **(B)**, and terpenoids **(C)**, standardized by Z-score in 5~8-year-old *Cinnamomum cassia*. Red indicates high accumulation, blue indicates low accumulation, and the right side of the heat map shows the classification of DAMs.

There were 72, 146, and 130 DAMs related to phenylpropanoids, flavonoids, and terpenoids were screened from 5~8-year-old *C*. *cassia*. The intensity of different metabolites in 5*~*8-year-old cinnamon was standardized, and the heatmap was drawn. Among these, the DAMs related to phenylpropanoid substances were divided into five subtypes: phenylpropanoic acids, hydroxycinnamic acids and derivatives, coumarins and derivatives, benzoic acids and derivatives and cinnamaldehydes. Coumarin and hydroxycinnamic acid had the largest accumulation in 8-year-old *C*. *cassia*, while phenylpropionic acid, benzoic acid, and cinnamaldehyde had the largest accumulation in 6-year-old *C*. *cassia* (**Figure 2A**). The DAMs related to flavonoids were divided into flavanol, flavanone, anthocyanidin, flavone, flavonol, and isoflavone, and most of them accumulated in 6-year-old *C*. *cassia* (**Figure 2B**). A total of 130 terpenoid-related DAMs were divided into 5 subtypes. Most terpenoids had the largest accumulation in 5-year-old *C*. *cassia*. All tetraterpenoids had the largest accumulation in 5-year-old *C*. *cassia* (**Figure 2C**).

### 3.3 Transcriptome sequencing analysis

Transcriptome sequencing of *C*. *cassia* samples from four growth years yielded a total of 526.03 million clean reads. After removing some low-quality sequences, 509.32 million clean reads were obtained, with a total base number of 76.4 Gb. The sequencing data quality evaluation results showed that the Q30 of each sample was ≥ 92.52% (**Supplementary Table S2**), which indicated that the sequencing results were reliable and could be analyzed in the next step. After assembling clean reads using Trinity software, a total of 131372 Unigenes were obtained and the average length of these Unigenes is 1113nt. Among these unigenes, 31,988 unigenes were 200–300 nt in length, and 99,384 unigenes were longer than 300 nt (**Supplementary Figure S2**).

### 3.4 Analysis of DEGs in 5–8-year-old *C. cassia*

**Figure 3:**
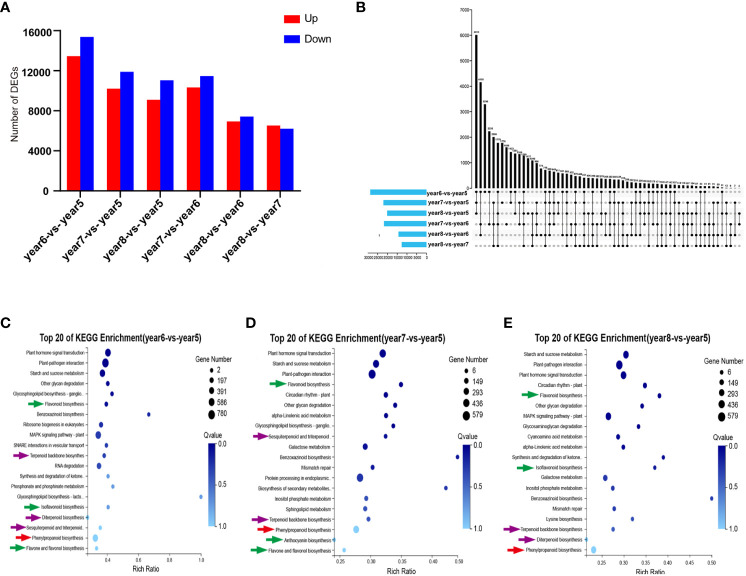
Statistical analysis of transcriptome of 5~8-year-old *C. cassia*. **(A)** The number of DEGs upregulated and downregulated in each group after comparing 5~8-year-old *C. cassia* in pairs. **(B)** The Upstet Plot set diagram of DEGs in each group after comparing 5~8-year-old *C. cassia* in pairs. The left histogram shows the total number of DEGs included in each group comparison. The lower part of intersection point represents the corresponding comparison group on the left, and the bar graph on the top represents the amount of DEGs shared under the intersection condition. **(C)** KEGG enrichment analysis of DEG in year6-*vs.*-year5. **(D)** KEGG enrichment analysis of DEGs in year7-*vs.*-year5. **(E)** KEGG enrichment analysis of DEGs in year8-*vs.*-year5.

A total of 44,455 DEGs were screened by comparing 5~8-year-old samples in pairs. In year6-*vs.*-year5, year7-*vs.*-year5, year8-*vs.*-year5, year7-*vs.*-year6, year8-*vs.*-year6, and year8-*vs.*-year7, 28,837, 22,099, 20,144, 21,794, 14,354, and 12,721 DEGs, respectively, were counted. Among them, except for year8-*vs.*-year7, there were more downregulated genes than upregulated genes in other comparison groups (**Figure 3A**). The Upstet Plot set diagram directly showed the distribution of DEGs in each comparison group. Among them, there were 411 DEGs in common among the 6 comparison groups, and 3,296, 1,776, 1,177, 1,606, 774, and 466 unique DEGs in the 6 comparison groups. In general, most DEGs were found in the year6-*vs.*-year5 comparison group (**Figure 3B**). Getorf 6.5.7.0 was used to predict the ORF of unigenes, and hmmsearch 3.0 was used to compare them with the TF protein domain. A total of 2641 TF coding genes belonging to 56 TF families were detected. Among them, the six families with the largest number of TFs were MYB (282), C2H2 (274), bHLH (197), C3H (146), ERF (141), and NAC (120). The 44,455 DEGs were compared with the 2641 TF-coding genes. A total of 53 TF families were identified, including 1,588 differentially expressed TFs. The six families with the most TFs were C2H2, MYB, bHLH, ERF, NAC, and C3H, with 167, 152, 134, 93, 83, and 81 TFs, respectively (**Supplementary Table S3**). In order to verify the reliability of the transcriptome data, 12 DEGs related to phenylpropanoid biosynthesis, flavonoid biosynthesis, and terpenoid biosynthesis were selected for RT-qPCR validation. The RT-qPCR results were consistent with the expression patterns in the RNA-seq analysis. This indicated that the results of RNA-seq analysis have high repeatability and reliability (**Supplementary Figure S3**).

KEGG pathway enrichment analysis was performed on DEGs selected from comparison groups of year6-*vs.*-year5, year7-*vs.*-year5, and year8-*vs.*-year5. The results indicated that DEGs in the three groups were mainly concentrated in three biosynthesis pathways: phenylpropanoid biosynthesis, flavonoid biosynthesis, and terpenoid backbone biosynthesis (**Figures 3C–E**). KEGG enrichment results of transcriptome data were consistent with KEGG enrichment results of metabolic data.

### 3.5 Integrative analysis of metabolome and transcriptome of phenylpropanoid biosynthesis

**Figure 4:**
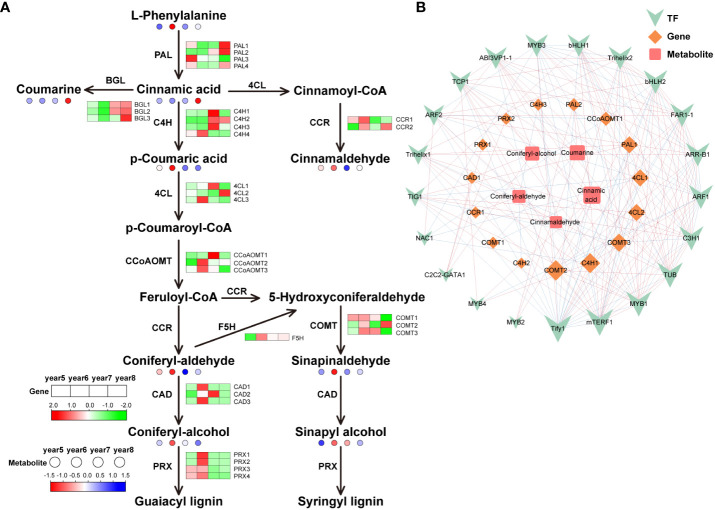
Integrative analysis of transcriptome and metabolome of the phenylpropanoid biosynthetic pathway in 5~8-year-old *C. cassia*. **(A)** Phenylpropanoid biosynthesis pathway constructed with DAMs and DEGs. Red and green boxes represent structural genes with upregulated and downregulated expression, respectively, while red and blue dots represent metabolites with upregulated and downregulated accumulation, respectively. **(B)** Correlation network diagram of phenylpropanoids. Among them, the high positive correlation is connected by red lines, and the high negative correlation is connected by blue lines. The size of the icon represents the number of genes, metabolites, and transcription factors that are highly correlated with it. The larger the icon, the more relevant substances.

To understand the differences of key genes expression levels and metabolites content in phenylpropanoid biosynthesis, flavonoid biosynthesis, and terpenoid biosynthesis during the development of *C*. *cassia*, heat maps were used to visually display the expression patterns of metabolites and genes in 5*~*8-year-old *C*. *cassia*. In the phenylpropanoid pathway, 30 genes encoding phenylpropanoid biosynthesis-related enzymes were identified. It included 4 cinnamate 4-hydroxylase (*C4H*), 4 phenylalanine ammonia-lyase (*PAL*), 4 peroxidase (*PRX*), 3 caffeoyl-CoA O-methyltransferase (*CCoAOMT*), 3 beta-glucosidase (*BGL*), 3 caffeic acid 3-O-methyltransferase (*COMT*), 3 4-coumarate-CoA ligase (*4CL*), 3 cinnamyl-alcohol dehydrogenase (*CAD*), 2 cinnamoyl-CoA reductase (*CCR*), and 1 ferulic acid-5-hydroxylase (*F5H*). Cinnamaldehyde is the main active component of cinnamon, and the accumulation of cinnamaldehyde reached the highest level in 6-year-old *C*. *cassia*, which may cause by the high expression of *CCR1*. At the same time, *PAL1* and *PAL2* were highly expressed in 8-year-old *C*. *cassia*, and the downstream metabolite cinnamic acid also reached maximum accumulation in 8-year-old *C*. *cassia*. In the lignin synthesis pathway, 6-year-old *C*. *cassia* had the highest product accumulation, and *C4H4*, *4CL3*, *CCoAOMT2*, *CCoAOMT3*, *CAD1*, *CAD3*, *F5H*, *PRX1*, *PRX2*, *PRX3*, *PRX4*, *COMT1*, and *COMT3* showed similar change patterns (**Figure 4A**). In the diagram of the regulatory network, 21 TFs, 15 DEGs, and 5 DAMs related to phenylpropanoid biosynthesis were highly correlated. Among them, *CCR1* has a positive regulatory effect on cinnamaldehyde synthesis, and TF *Tify1* was significantly related to most metabolites, genes, and TFs (**Figure 4B**).

### 3.6 Integrative analysis of metabolome and transcriptome of flavonoid biosynthesis

**Figure 5:**
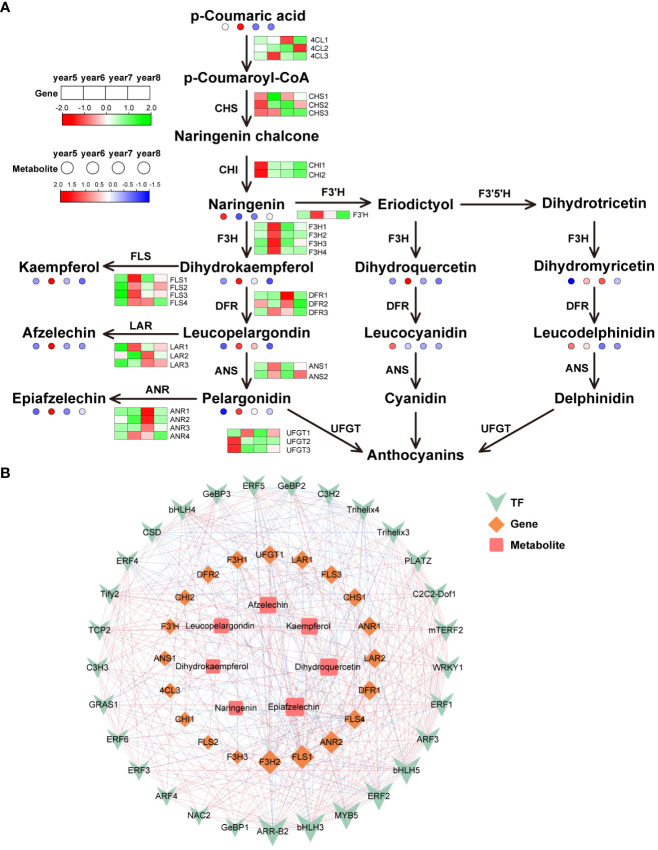
Integrative analysis of transcriptome and metabolome of the flavonoids biosynthetic pathway in5~8-year-old *C. cassia*. **(A)** Flavonoids biosynthesis pathway constructed with DAMs and DEGs. **(B)** Correlation network diagram of flavonoids.

There were 32 key synthetase genes in the flavonoid biosynthesis pathway: 4 Flavanone 3-hydroxylase (*F3H*), 4 flavonol synthase (*FLS*), 3 chalcone synthase (*CHS*), 3 bifunctional dihydroflavonol 4-reductase (*DFR*), 3 *4CL*, 3 leucoanthocyanidin reductase (*LAR*), 4 anthocyanidin reductase (*ANR*), 2 anthocyanidin synthase (*ANS*), 2 chalcone isomerase (*CHI*), 3 flavonol 3-O-glucosyltransferase (*UFGT*) and 1 flavonoid 3’-hydroxylase (*F3’H*). Most flavonoid metabolites were highest in 6-year-old *C*. *cassia*, which is consistent with the metabolome results and its gene expression pattern (**Figure 5A**). Among them, the high expression of *F3H* and *FLS* in 6-year-old *C*. *cassia* made dihydrokaempferol and kaempferol accumulate the highest. In addition, 29 TFs, 20 DEGs, and 7 differentially expressed metabolites constituted a diagram of the regulatory network. *F3H2* and *FLS1* had the strongest correlations with DAMs and TFs. They were positively correlated with metabolites afzelechin, kaempferol, epiafzelechin, and dihydroquercetin and negatively correlated with most TFs. This indicates that *F3H2* and *FLS1* may play a crucial role in regulating the synthesis of flavonoid metabolite. In addition, the correlation between transcription factor *ERF2* and *F3H2* and *FLS1* is high, indicating that *ERF2* may participate in the synthesis of flavonoids by affecting the expression of *F3H2* and *FLS1*, which needs further validation (**Figure 5B**).

### 3.7 Integrative analysis of metabolome and transcriptome of terpenoid biosynthesis

**Figure 6:**
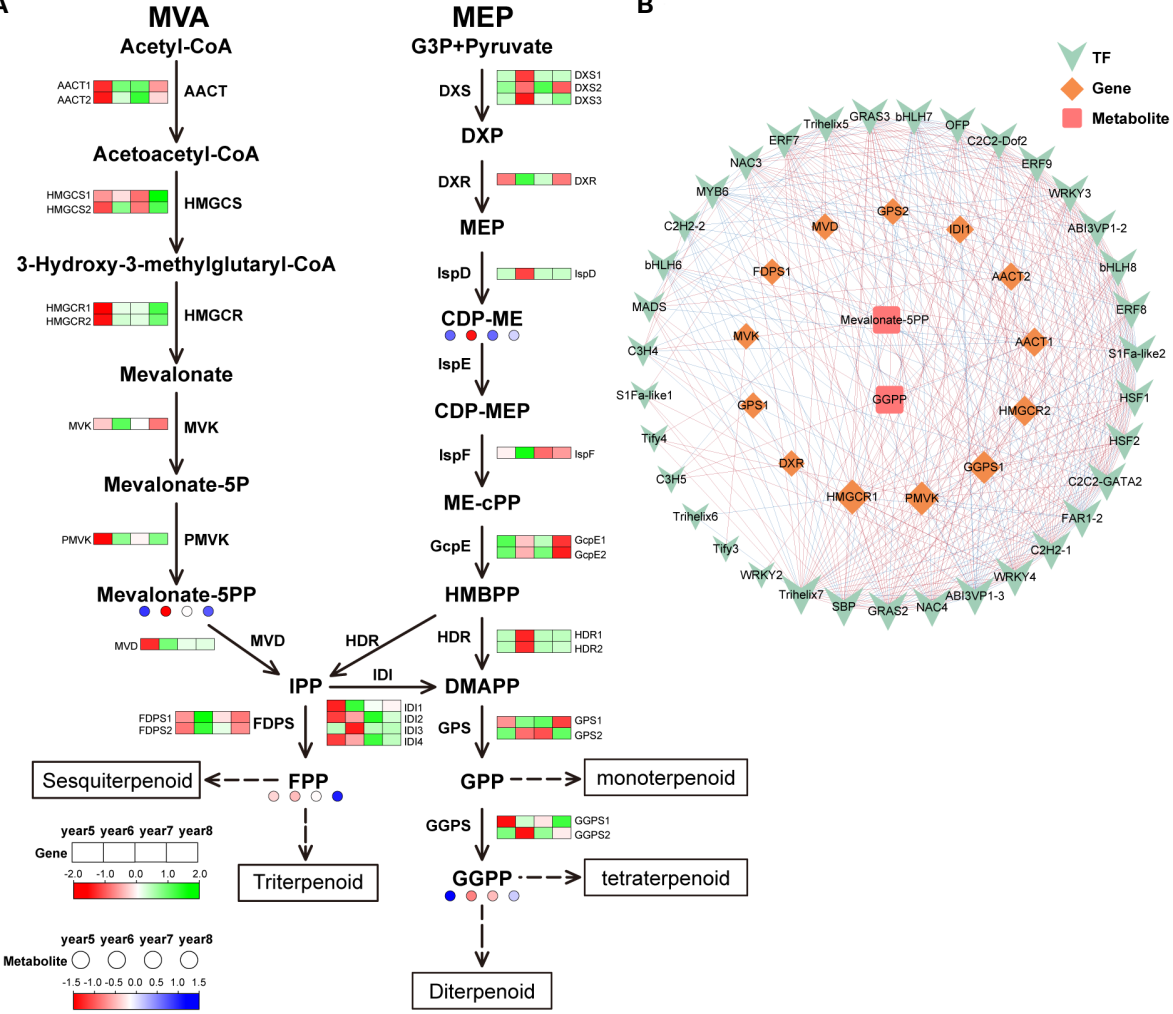
Integrative analysis of transcriptome and metabolome of terpenoid biosynthetic pathway in 5~8 years old *C. cassia*. **(A)** Terpenoid biosynthesis pathway constructed with DAMs and DEGs. **(B)** Correlation network diagram of terpenoid.

29 DEGs were discovered in the terpenoid biosynthesis pathway: 4 isopentenyl-diphosphate Delta-isomerase (*IDI*), 3 1-deoxy-D-xylulose-5-phosphate synthase (*DXS*), 1 mevalonate kinase (*MVK*), 2 farnesyl diphosphate synthase (*FDPS*), 1 2-C-methyl-D-erythritol 2,4-cyclopyrophosphate synthetase (*IspF*), 1 diphosphomevalonate decarboxylase (*MVD*), 1 2-C-methyl-D-erythritol 4-phosphate cytidylyltransferase (*IspD*), 2 hydroxymethylglutaryl-CoA synthase (*HMGCS*), 1 1-deoxy-D-xylulose-5-phosphate reductoisomerase (*DXR*), 2 (E)-4-hydroxy-3-methylbut-2-enyl-diphosphate synthase (*GcpE*), 1 phosphomevalonate kinase (*PMVK*), 2 geranylgeranyl diphosphate synthase, type II (*GGPS*), 2 4-Hydroxy-3-methylbut-2-enyl diphosphate reductase (*HDR*), 2 acetyl-CoA C-acetyltransferase (*AACT*), 2 hydroxymethylglutaryl-CoA reductase (*HMGCR*), and 2 geranyl diphosphate synthase (*GPS*). Isoopentenyl diphosphate (IPP) can be synthesized by two routes: MVA pathway and MEP pathway. In the MVA pathway, most of the DEGs were highly expressed in 5-year-old *C*. *cassia*, which was corresponds to the results of the metabolomics analysis. In the MEP pathway, *DXS* and *IspD* genes were highly expressed in 6-year-old cinnamon, which regulates the massive accumulation of 4-(Cytidine 5’-diphospho)-2-C-methyl-D-erythritol (CDP-ME) in 6-year-old *C*. *cassia*. In addition, the high expression of *HDR* and *GGPS2* in 6-year-old *C*. *cassia* further promoted the accumulation of downstream GGPP (geranylgeranyl diphosphate) (**Figure 6A**). In the terpenoid biosynthesis pathway, 35 TFs, 13 DEGs, and 2 differentially expressed metabolites together constituted the diagram of the regulatory network. The metabolite Mevalonate-5PP was significantly negatively correlated with *FDPS1* and *AACT2*, which was corresponds to the results in the terpenoid biosynthesis pathway. The transcription factor *Trihelix5* was significantly positively correlated with gene *AACT2*, indicating that Trihelix5 may regulate the synthesis of metabolite Mevalonate 5PP by affecting gene *AACT2*. In conclusion, terpenoid skeleton synthesis, transcription of structural genes, and TF regulation were significantly related (**Figure 6B**).

## 4 Discussion

In *C*. *cassia*, phenylpropanoids, flavonoids, and terpenoids determine its medicinal value and edible quality. In the different growth and development stages of medicinal plants, transcriptional reprogramming and the redirection of metabolic flux occur in a variety of biosynthetic pathways (Liu et al., 2017). Many studies have found that the growth years can significantly affect the accumulation of effective components in plant (Geng et al., 2011; Li et al., 2013). Similar to the results of the metabolome data, DEGs in *C*. *cassia* were significantly enriched in phenylpropanoid, flavonoid and terpenoid biosynthetic pathways at different growth years (**Supplementary Figure S4**), indicating that the change in metabolite accumulation patterns was strictly controlled by DEGs.

Phenylpropanoid biosynthesis starts with the early evolution of freshwater algae to terrestrial plants. At present, phenylpropanoid biosynthesis in terrestrial plants has evolved through a variety of branch pathways. PAL is a key enzyme and rate-limiting enzyme connecting primary metabolism and phenylpropanoid biosynthesis, which catalyzes L-phenylalanine to produce trans-cinnamic acid, lignin, coumarin, cinnamaldehyde, and other metabolites (Jiao et al., 2020). As an intermediate product, trans cinnamic acid can be further converted into lignin, coumarin, cinnamaldehyde, and other metabolites. The content of coumarin was highest in 8-year-old *C*. *cassia*, which is basically consistent with the expression trend of three *BGLs* (**Figure 4A**), indicating that coumarin synthesis is under the control of these three *BGLs*. Cinnamaldehyde has antibacterial (Vijayan and Mazumder, 2018), anti-tumor (Koppikar et al., 2010), and other activities. Geng et al. (2011) used GC-MS technology to detect and analyze the content of cinnamaldehyde in cinnamon oil extracted from 5*~*12-year-old cinnamon and found that the content was the highest in 6-year-old *C*. *cassia.* Gao et al. (2023) analyzed the differences of genes and metabolites in different *C*. *cassia* tissues through transcriptome and metabolomics. They found that cinnamaldehyde content in *C*. *cassia* bark was higher than that in branches and leaves, and CCR gene content was also higher in *C*. *cassia* bark, which was corresponded to the results of our study. Therefore, we speculated that this was due to the high expression levels of the *CCR* in 6-year-old *C*. *cassia* (**Figure 4A**). In the branching pathway to lignin, the expression of most genes in 6-year-old *C*. *cassia* was high, and the expression of the *C4H4* gene was the highest, which was consistent with the accumulation of p-coumaric acid. It was inferred that *C4H4* had strong competitiveness for substrates, resulting in the massive production of p-coumaric acid.

The basic structure of flavonoids is C6-C3-C6, and its synthesis pathway is the branch with the most kinds of metabolites in the phenylpropanoid biosynthesis pathway (Stobiecki and Kachlicki, 2006). This pathway is relatively conservative in plant evolution, and the steps of flavonoid synthesis in most plants are the same. According to the RNA-seq map, 32 DEGs related to flavonoid synthesis were identified. *4CL*, *CHS, F3H, CHI* and *F3’H* regulate the synthesis of early precursors of flavonoids, *DFR*, *ANS*, and *UFGT* regulate anthocyanin synthesis, *FLS* regulates flavonol synthesis, and *LAR* and *ANR* are related to flavanol synthesis. The expression levels of most of these genes was the highest in 6-year-old *C*. *cassia*, which was consistent with the accumulation of flavonoids and their derivatives (**Figure 5A**). F3H is the center of the whole flavonoid metabolic pathway, which can catalyze flavanone to generate dihydroflavonol, dihydroquercetin, and dihydromyricetin. These dihydroflavonols are important intermediates in the synthesis of flavonol, flavanol, and anthocyanin (Holton and Cornish, 1995). FLS uses dihydroflavonol as the substrate to form flavonol compounds (Forkmann and Martens, 2001). Xu et al. (2012) cloned the gene *GbFLS* from *Ginkgo biloba L*. into the pET-28a (+). Then, transformed recombinant plasmid into *Escherichia coli* BL21 (DE3). The enzyme activity test results indicated that the recombinant GbFLS protein expressed *in vitro* catalyzes dihydrokaempferol to generate kaempferol and simultaneously catalyzes naringen to convert kaempferol. This study showed that GbFLS is a multifunctional dioxygenase. In the pathway map, all members of the *F3H* and *FLS* gene families showed the highest expression in 6-year-old *C*. *cassia* (**Figure 5A**). It is speculated that the high expression of key structural genes *F3H* and *FLS* in the flavonoid biosynthesis pathway of *C*. *cassia* led to the mass synthesis of flavonoids.

Terpenoids are important secondary metabolites of *C*. *cassia*. They are mainly synthesized in two ways: MVA pathway and MEP pathway (**Figure 6A**). The main difference between the two synthesize pathways is that the synthesis mechanism and final products of the intermediate IPP (isopentenyl pyrophosphate) and the DMAPP (isomer dimethyl allyl pyrophosphate) are different. IPP and DMAPP are common precursors of all terpenoids. The MVA pathway in cytoplasm uses acetyl CoA as a raw material to produce IPP, while the MEP pathway in plastids uses pyruvic acid and glyceraldehyde-3-phosphate as raw materials to form IPP and DMAPP (Vranová et al., 2013). IPP generated by MVA pathways and MEP pathway can pass through the plastid membrane and be used by each other (Zhang et al., 2022). According to the metabolomic data, most terpenoids had the largest accumulation in 5-year-old *C*. *cassia* (**Figure 6A**). Therefore, we speculated that the mechanism of IPP formation was different in *C*. *cassia* with different ages. In the MVA pathway, *MVD*, which regulates IPP synthesis, is highly expressed in 5-year-old *C*. *cassia*. In the MEP pathway, *HDR* regulating IPP synthesis has the highest expression in 6-year-old *C*. *cassia*. Through the KEGG pathway annotation results, we screened 29 DEGs related to terpenoid biosynthesis and further analyzed the expression levels of these genes. Most of the structural gene expression patterns on the MVA pathway corresponded to the metabolome data results (**Figure 6A**), indicating that the biosynthesis of terpenoid compounds in *C*. *cassia* might be more dependent on the MVA pathway. Schramek et al. (2014) found that the biosynthesis of ginsenoside, a typical terpenoid compound in *Radix Ginseng*, mainly through the MVA pathway by using a^13^CO_2_ pulse chase technology. Green *Zanthoxylum armatum* and red *Z*. *armatum* differ in flavor and aroma because the terpenoids in green *Z*. *armatum* are synthesized through MVA and MEP, while the terpenoids in red *Z*. *armatum* are mainly produced through MEP (Fei et al., 2021). In general, terpenoid biosynthesis in plants can depend on a certain pathway, but the MVA pathway and MEP pathway can also compensate for each other to ensure the normal growth of plants.

TFs activate or inhibit the co-expression of multiple genes by specifically binding to the DNA sequence of the regulatory region (Dare et al., 2008). The correlation network between transcriptome and metabolome can be used to clarify functional relationships between genes and metabolites. It can also use to identify key TFs. This study determined the Pearson correlation coefficient of TFs, DEGs, and DAMs related to the synthesis of phenylpropanoids, flavonoids, and terpenoids and excavated the core regulatory network (**Figures 4B**, **5B**, **6B**). A high correlation between specific DEGs, TFs, and metabolites indicates that these structural genes/TFs play an important role in the growth and development of *C*. *cassia*. The analysis of *C*. *cassia* transcript libraries in different growth years showed that 1,588 TFs had different expression levels (**Supplementary Table S3**). During the development of plant, TFs play an important role in regulating the production of effective substances, including positive and negative regulation. *TmMYB3* (Yu et al., 2020), *PpNAC1* (Jin et al., 2022), and *VqWRKY31* (Yin et al., 2022) have been proven to increase substance synthesis by promoting the expression of structural genes. TFs can also be expressed in tissues as repressors to prevent ectopic substances accumulation. Some repressors, such as *PtrMYB57*, can form MBW complexes with other TFs to reduce substance production (Wan et al., 2017). However, some TFs have dual functions, acting as inhibitors and activators (Chen et al., 2021). We identified some TFs highly related to the synthesis of phenylpropanoids, flavonoids, and terpenoids through co-expression network analysis, such as *MYBs*, *ERFs*, *bHLHs*, *NACs*, and *WRKYs* (**Figures 4B**, **5B**, **6B**). Previous studies have isolated and identified some TFs that play positive and negative regulatory roles in the of phenylpropanoid and flavonoid in plants. For example, *MYB165* was negatively correlated with various genes in flavonoid and phenylpropanoid biosynthesis pathways in *Populus L*. (Ma et al., 2018). Using yeast hybridization, three *ERF* TF family members have been shown to regulate the synthesis of citrus flavonoids by regulating type IV chalcone isomerase (Zhao et al., 2021). *MsMYB* directly binds to the cis-acting regulatory element of the large subunit of GPP synthetase (*MsGPPS LSU*) and negatively regulates terpenoid biosynthesis (Reddy et al., 2017). The spatiotemporal expression patterns of positive and negative regulators may determine the balance of the accumulation levels of active components in *C*. *cassia*. This study showed many candidate regulators with active components in *C*. *cassia*, and the investigators plan to further explore the regulatory mechanisms of these TFs in biosynthesis process of active components.

## 5 Conclusions

In our study, integrative analysis of metabolome and transcriptome were performed on 5~8-year-old *C*. *cassia* to understand the dynamic accumulation mechanism of active ingredients. The high levels expression of phenylpropanoid and flavonoid pathway genes in 6-year-old *C*. *cassia* led to significantly higher content of phenylpropanoids and flavonoids such as cinnamic aldehyde and coumaric acid in 6-year-old than in others. Through co-expression network analysis, genes and TFs were identified that regulate the biosynthesis and regulation of phenylpropanoids and flavonoids, and it was predicted that TFs such as *MYBs*, *bHLHs*, *ERFs*, *NACs*, and *WRKYs* were involved in the regulation of phenylpropanoids and flavonoids. In addition, metabolome analysis showed that the accumulation of terpenoids in 5-year-olds was significantly higher than in others, which was caused by high levels expression upstream genes in the terpenoid synthesis pathway. Together, this study provides new understanding for the accumulation and synthesis of phenylpropanoids, flavonoids, and terpenoids in *C*. *cassia*, which also lays a solid biological foundation for the breeding of high-quality *C*. *cassia*.

## Data availability statement

The original contributions presented in the study are included in the article/**Supplementary Material**. Further inquiries can be directed to the corresponding author.

## Author contributions

HG: Conceptualization, Data curation, Formal analysis, Funding acquisition, Investigation, Methodology, Writing – original draft, Writing – review & editing. MS: Data curation, Investigation, Visualization, Writing – review & editing. HZ: Investigation, Visualization, Writing – review & editing. HS: Data curation, Visualization, Writing – review & editing. QY: Funding acquisition, Project administration, Supervision, Writing – review & editing.
