# Supplementary material for: Integrated metabolomic and transcriptomic analyses revealed metabolite variations and regulatory networks in Cinnamomum cassia Presl from four growth years
Source: Front Plant Sci. 2024 Jan 10;14:1325961. doi: 10.3389/fpls.2023.1325961 (PMC10806117; doi:10.3389/fpls.2023.1325961)
Supplement: Supplementary file 1 [file DataSheet_1.docx]

Supplementary Material

| Gene ID | Forward primer (5'-3') | Reverse primer (5'-3') |
| --- | --- | --- |
| CL987.Contig19 | GGGTAGTCGTAGTCTCATCTAT | CACGTTGGACAGAGACTTATT |
| CL4825.Contig4 | AGACAACTGAGAACTGGTATTG | AGCGGAGGACCAAGTATT |
| Unigene21677 | AGTGCAGAGCAACACAATC | GGCCGTCATCATCTTTAGTATC |
| CL2424.Contig3 | GCCGATGCCCTATCTTATTC | CAGTCATGGAGTATCCACTTC |
| CL10705.Contig2 | AGACCCTGTTAAGGTTGTTG | GCCTTGTCGTAGAAGTATGAG |
| CL3860.Contig3 | CCGTGTTGAGGAGTGTATAG | CCTCGAAGATGCTGAAGATAG |
| CL91.Contig1 | AGCTGGCCAAAGAGAATATG | GGATTGAAGGAAGCAGAGAAG |
| CL956.Contig7 | TCTAAGGGTGTTCACCTATCT | CAACCTGTCCAGTAGTCTTTC |
| Unigene6471 | CATTATACACGTCGGCAGAG | GTTGGGAGATGGAGCATAAG |
| CL11148.Contig18 | CCTTCAATATTCTCAGGCTCTATC | TATCGGGATTCGCCTCTATC |
| CL11523.Contig13 | CCCTTGGAGTCAATTCCTACAG | CCTTTAAGCAAGAGGGCATCTA |
| CL1972.Contig12 | TGGAAGGGAGTTGCTTATTAC | AAGTGCTTCAGGAAGATCATAG |
| Unigene85771 | GTACGCCGAAATAGAGGTTATG | TCACCTGCCTCCAAGTTA |
| CL4713.Contig3 | ATCCGAAGGCAAGAGAGT | TACTGGGTCAAGGTGGATAG |
| GAPDH | GTTGCTGTCTTTGGTCTCA | CAGCAGCCTTGTCCTTATC |

**Supplementary Table 1.** [Sequences of qPCR primers](https://xueshu.baidu.com/usercenter/paper/show?paperid=d5bbad151b86c378b8d91a76761f4ba5&site=xueshu_se).

| Sample | Total raw reads (M) | Total clean reads (M) | Total clean bases(Gb) | Clean reads Q20(%) | Clean reads Q30(%) | Clean reads ratio(%) |
| --- | --- | --- | --- | --- | --- | --- |
| year5_1 | 43.82 | 42.58 | 6.39 | 97.22 | 92.61 | 97.17 |
| year5_2 | 43.82 | 42.37 | 6.36 | 97.23 | 92.68 | 96.7 |
| year5_3 | 43.82 | 42.07 | 6.31 | 97.35 | 92.97 | 96.01 |
| year6_1 | 43.82 | 42.37 | 6.35 | 97.28 | 92.75 | 96.68 |
| year6_2 | 45.57 | 43.59 | 6.54 | 97.43 | 93.14 | 95.64 |
| year6_3 | 43.82 | 42.67 | 6.4 | 97.28 | 92.77 | 97.36 |
| year7_1 | 43.82 | 42.69 | 6.4 | 97.22 | 92.6 | 97.43 |
| year7_2 | 43.82 | 42.37 | 6.36 | 97.27 | 92.76 | 96.68 |
| year7_3 | 43.82 | 42.47 | 6.37 | 97.45 | 93.22 | 96.92 |
| year8_1 | 43.82 | 42.63 | 6.4 | 97.3 | 92.81 | 97.29 |
| year8_2 | 43.82 | 42.36 | 6.35 | 97.27 | 92.73 | 96.66 |
| year8_3 | 42.26 | 41.15 | 6.17 | 97.19 | 92.52 | 97.39 |

**Supplementary Table 2.** Quality statistics of reads in raw transcriptome data after filtering.

| TF Family | Number of Gene | TF Family | Number of Gene | TF Family | Number of Gene | TF Family | Number of Gene |
| --- | --- | --- | --- | --- | --- | --- | --- |
| ABI3VP1 | 58（28） | CPP | 27（14） | LOB | 18（10） | TCP | 33（22） |
| ERF | 141（93） | CSD | 7（4） | MADS | 40（23） | TIG | 41（39） |
| ARF | 103（78） | DBP | 2（0） | MYB | 282（152） | TUB | 36（24） |
| ARR-B | 26（17） | E2F-DP | 18（10） | NAC | 120（83） | Tify | 26（22） |
| Alfin-like | 23（9） | EIL | 5（3） | NOZZLE | 4（2） | Trihelix | 52（36） |
| BBR/BPC | 10（5） | FAR1 | 65（36） | OFP | 8（4） | ULT | 2（0） |
| BES1 | 16（12） | FHA | 31（14） | PBF-2-like | 3（0） | VARL | 8（4） |
| BSD | 9（7） | G2-like | 100（66） | PLATZ | 13（9） | VOZ | 7（5） |
| C2C2-CO-like | 8（7） | GRAS | 57（52） | RWP-RK | 12（7） | WRKY | 91（57） |
| C2C2-Dof | 50（38） | GeBP | 11（8） | S1Fa-like | 4（3） | Zn-clus | 118（40） |
| C2C2-GATA | 53（26） | HB | 12（9） | SBP | 37（21） | bHLH | 197（134） |
| C2H2 | 274（167） | HRT | 5（4） | SRS | 4（1） | bZIP | 66（29） |
| C3H | 146（81） | HSF | 27（15） | Sigma70-like | 6（5） | mTERF | 64（23） |
| CAMTA | 2（2） | LIM | 51（19） | TAZ | 5（5） | zf-HD | 7（4） |

**Supplementary Table 3.** Number of transcription factors and differentially expressed transcription factors. The numbers outside the brackets indicate the number of transcription factors in each family; The number of differentially expressed transcription factors in each family is indicated in parentheses.

**
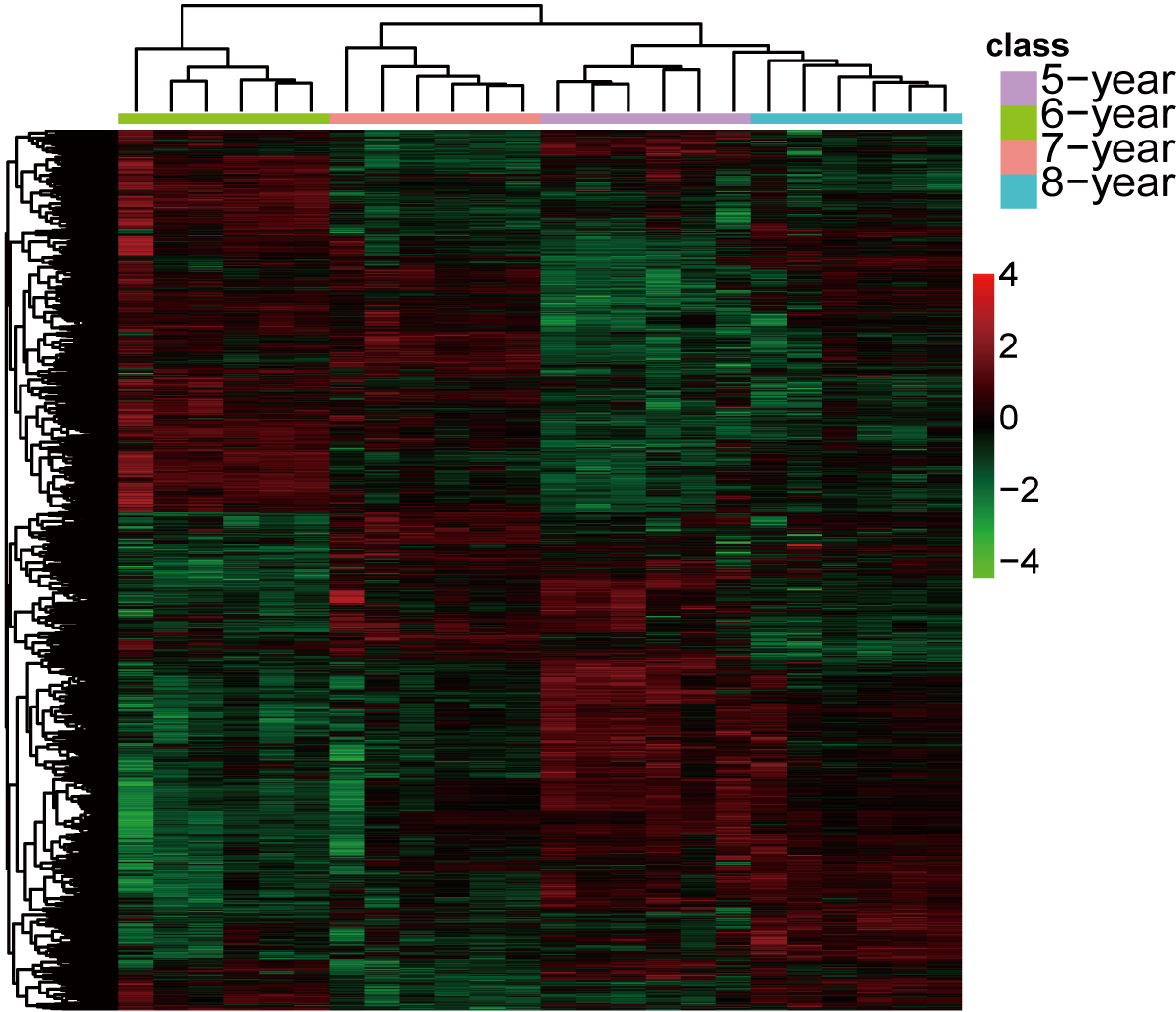
**

**Supplementary Figure 1.** Cluster analysis diagram of 5-year-old, 6-year-old, 7-year-old and 8-year-old *Cinnamomum cassia* Presl bark.


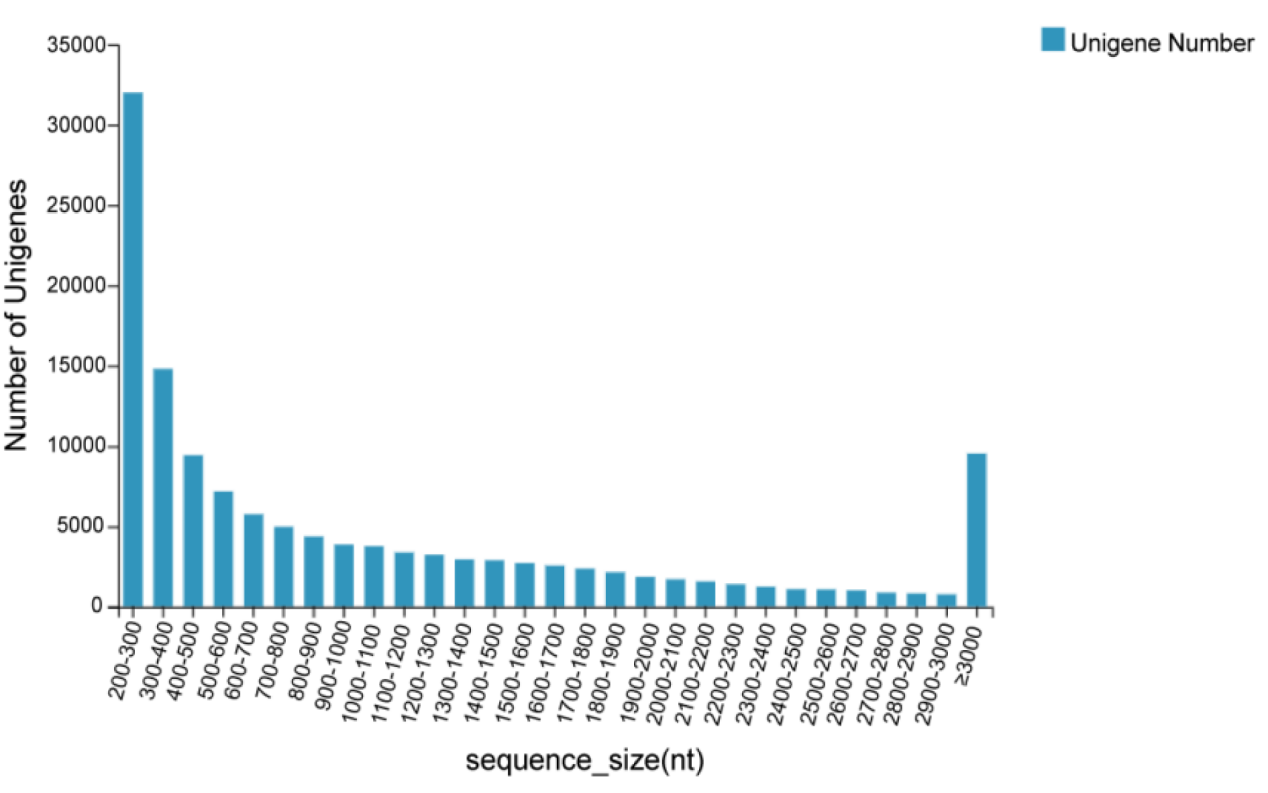


**Supplementary Figure 2.** Length distribution of Unigenes.


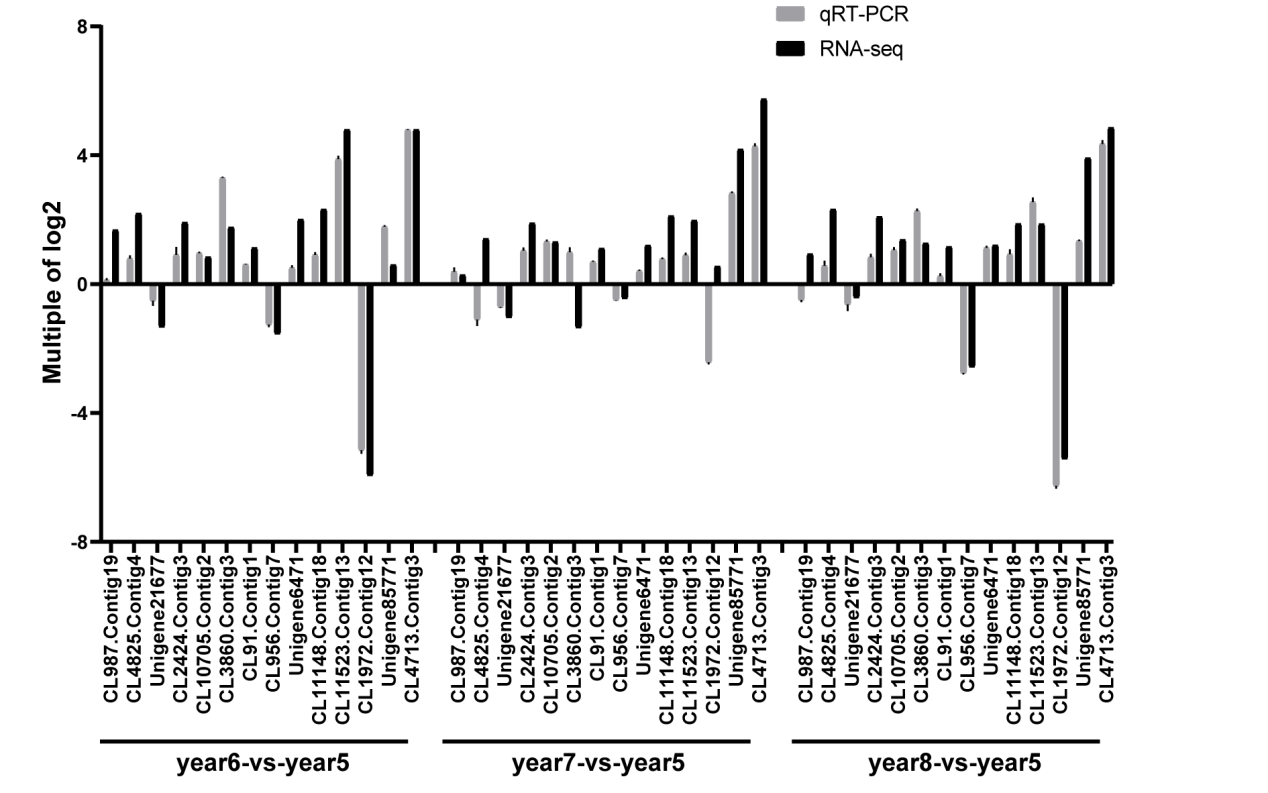


**Supplementary Figure 3.** Analysis of the expression of 14 genes in the main metabolic pathways of *C. cassia* bark.


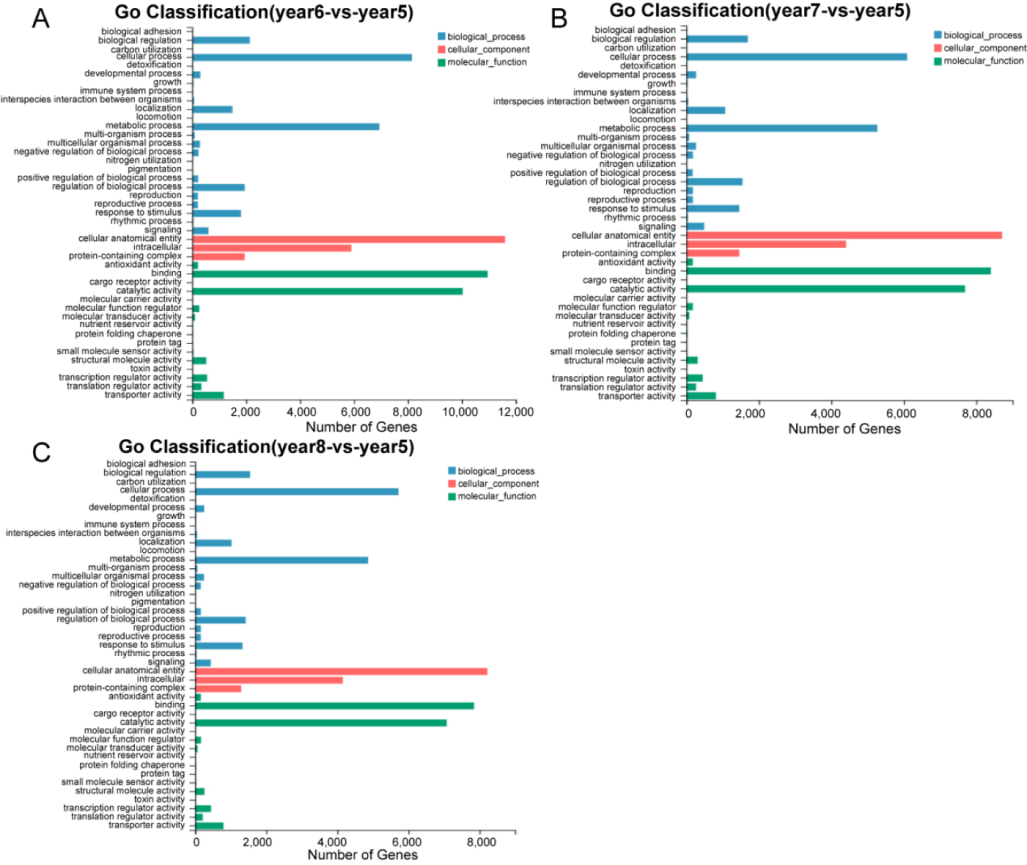


**Supplementary Figure 4.** GO enrichment analysis of screened differential genes with 5-year-old *C*. *cassia* bark as control. (A) Functional classification of differentially expressed genes in year6-vs-year5. (B) Functional classification of differentially expressed genes in year7-vs-year5. (C) Functional classification of differentially expressed genes in year8-vs-year5.
